# Clinical, Molecular and Genetic Validation of a Murine Orthotopic Xenograft Model of Pancreatic Adenocarcinoma Using Fresh Human Specimens

**DOI:** 10.1371/journal.pone.0077065

**Published:** 2013-10-18

**Authors:** Dustin M. Walters, Jayme B. Stokes, Sara J. Adair, Edward B. Stelow, Cheryl A. Borgman, Bryce T. Lowrey, Wenjun Xin, Edik M. Blais, Jae K. Lee, Jason A. Papin, J. Thomas Parsons, Todd W. Bauer

**Affiliations:** 1 Department of Surgery, University of Virginia, Charlottesville, Virginia, United States of America; 2 Department of Pathology, University of Virginia, Charlottesville, Virginia, United States of America; 3 Department of Public Health Sciences, University of Virginia, Charlottesville, Virginia, United States of America; 4 Department of Biomedical Engineering, University of Virginia, Charlottesville, Virginia, United States of America; 5 Department of Microbiology, Immunology and Cancer biology, University of Virginia, Charlottesville, Virginia, United States of America; University of Nebraska Medical Center, United States of America

## Abstract

**Background:**

Relevant preclinical models that recapitulate the key features of human pancreatic ductal adenocarcinoma (PDAC) are needed in order to provide biologically tractable models to probe disease progression and therapeutic responses and ultimately improve patient outcomes for this disease. Here, we describe the establishment and clinical, pathological, molecular and genetic validation of a murine, orthotopic xenograft model of PDAC.

**Methods:**

Human PDACs were resected and orthotopically implanted and propagated in immunocompromised mice. Patient survival was correlated with xenograft growth and metastatic rate in mice. Human and mouse tumor pathology were compared. Tumors were analyzed for genetic mutations, gene expression, receptor tyrosine kinase activation, and cytokine expression.

**Results:**

Fifteen human PDACs were propagated orthotopically in mice. Xenograft-bearing mice developed peritoneal and liver metastases. Time to tumor growth and metastatic efficiency in mice each correlated with patient survival. Tumor architecture, nuclear grade and stromal content were similar in patient and xenografted tumors. Propagated tumors closely exhibited the genetic and molecular features known to characterize pancreatic cancer (e.g. high rate of *KRAS*, *P53*, *SMAD4* mutation and EGFR activation). The correlation coefficient of gene expression between patient tumors and xenografts propagated through multiple generations was 93 to 99%. Analysis of gene expression demonstrated distinct differences between xenografts from fresh patient tumors versus commercially available PDAC cell lines.

**Conclusions:**

The orthotopic xenograft model derived from fresh human PDACs closely recapitulates the clinical, pathologic, genetic and molecular aspects of human disease. This model has resulted in the identification of rational therapeutic strategies to be tested in clinical trials and will permit additional therapeutic approaches and identification of biomarkers of response to therapy.

## Introduction

Pancreatic ductal adenocarcinoma (PDAC) is an insidious disease, with the shortest survival of any solid malignancy [1]. Surgical resection offers some patients a possibility of cure, but the vast majority of patients have unresectable, locoregionally advanced or metastatic disease at diagnosis. For these patients, medical therapy only marginally prolongs survival [2]. Even after potentially curative resection, long-term survival is rare [3] due to ineffective adjuvant therapy. Thus, in order to improve outcomes, more effective therapies are needed, as well as better appreciation of therapeutic resistance mechanisms. Integral to this is the development and utilization of well validated preclinical models that reflect the pathological, molecular and cellular properties of human tumors.

Accordingly, various preclinical models have been established to study PDAC, ranging from simple *in vitro* cell culture models to whole animal *in vivo* models. *In vitro* models offer advantages such as efficient derivation of data, control over drug delivery, lower cost and reproducibility of results, allowing for high-throughput analysis of multiple cell lines. However, two-dimensional culture poorly recapitulates *in vivo* biologic behavior and drug delivery, and undermines the impact of the tumor microenvironment. Thus, we have demonstrated in our laboratory that *in vitro* assays do not correlate with response in the orthotopic xenograft model for targeted therapies to focal adhesion kinase (FAK) [4], urokinase plasminogen activator receptor (uPAR) [5], and EGFR/Her2 and MEK [6].

Due to these limitations, several *in vivo* models have been developed [7], including genetically engineered murine models which have been engineered with mutations in *KRAS* plus deletions or mutations in *P53* [8], *P16INK4* [9], *MIST* [10], *SMAD4* [11], or *TGFβ* [12]. The tumors generated often progress from pancreatic intraepithelial neoplasia (PanIN) lesions to invasive cancer, similar to the progression sequence in humans. Unfortunately, these models frequently have incomplete penetrance, long latency periods, and variable metastasis. Tseng et al [13] have developed an orthotopic model of murine pancreatic cancer using immunocompetent mice. This model benefits from an intact immune system, but like all other genetically engineered mouse models of pancreatic cancer, is limited to specific, predefined genetic mutations and thus poorly reflects the genetic diversity of human PDAC.

Invasive approaches have been used in murine models of PDAC, where cells or tumor pieces are implanted heterotopically (subcutaneous) [14] or orthotopically (intrapancreatic) [15]. Subcutaneous models are attractive because of their technical ease and straightforward assessment of tumor size by caliper measurement, useful for determining drug response. These models, however, poorly recapitulate the pancreatic tumor microenvironment, which likely plays a significant role in tumor cell behavior. For instance, in the subcutaneous xenograft model using implantation of human PDAC described by Rubio-Viqueira et al [14], the correlation between human tumor and xenograft tumor gene expression was determined for 15 selected genes and only three genes exhibited statistically significant correlation, suggesting that the subcutaneous model was not accurately reflecting tumor cell signaling in patients. An additional limitation of subcutaneous xenograft models is the lack of peritoneal and liver metastases, thus precluding this as an endpoint for therapy.

While each of these models has potential value, we believe that orthotopic xenograft models are the best for studying novel therapeutic approaches to PDAC because such models more closely recapitulate human disease [7]. Many xenograft models utilize high-passage, commercially available pancreatic cancer cells lines. Unfortunately, because these cell lines have undergone selective pressure and genetic drift during years to decades of *in vitro* cell culture, they often are not representative of their original human source tumor [16]. More sophisticated models using freshly-derived human specimens have been described; however, these models have not validated the growth behavior and genetic/molecular signature of xenografted tumors with patient survival and genetic/molecular signaling of the patient tumor [17]. Herein, we describe an orthotopic xenograft model using implantation of fresh human pancreatic cancer specimens and detail an extensive pathologic, genetic and molecular characterization of the tumors and correlation to the patient tumors and patient survival.

## Materials and Methods

### Ethics Statement

Collection of human PDAC specimens was performed with approval of the Institutional Review Board at the University of Virginia in coordination with the Biorepository and Tissue Research Facility. All patients provided written consent for participation and no patients received neoadjuvant therapy. This study was carried out in strict accordance with the recommendations in the Guide for the Care and Use of Laboratory Animals of the National Institutes of Health [18]. The protocol was approved by the Animal Care and Use Committee of the University of Virginia (PHS Assurance #A3245-01).

### Acquisition of Patient Tumors and Orthotopic Implantation into Mice

**Figure 1:**
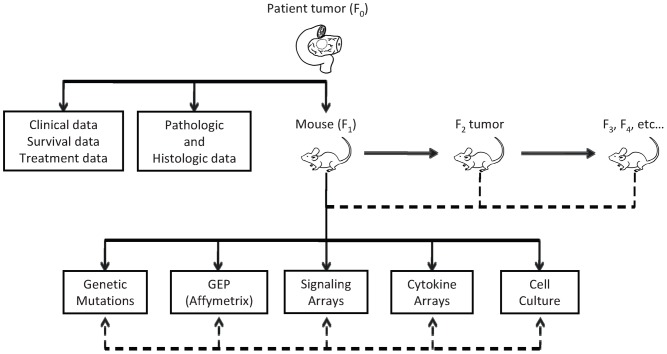
Overall model schema. After resection, human pancreatic ductal adenocarcinomas are orthotopically implanted into the pancreases of immunocompromised mice and propagated in subsequent generations. Tumors undergo genetic and proteomic assessment. Clinical and pathologic data are collected for each individual human tumor.

Following resection and pathological review of the patient tumor, residual tumor tissues were collected and placed in Roswell Park Memorial Institute media (RPMI) for surgical transplantation (below) or cryopreservation in fetal bovine serum (FBS) with 10% DMSO (Figure 1). Six to eight week old, male, non-obese, diabetic, severe combined immunodeficient (NOD SCID) and athymic nude mice (National Cancer Institute, Fredricksburg, MD) were used. To achieve more efficient engraftment during initial establishment of the human PDAC tumor line, NOD SCID mice were used for F_1_ and F_2_ generations. For propagation of the tumor line beyond these first two generations, athymic nude mice were used, as they retain innate immunity (natural killer cells, B lymphocytes, antigen presenting cells, and complement activity), which is impaired in NOD SCID mice. Mice were housed in pathogen-free conditions, acclimated to their new surroundings for at least 48 hours prior to tumor engraftment, and maintained in accordance with institutional standards. All animal surgery was performed under 2,2,2-tribromoethanol anesthesia (4 mg/10 gm body weight). Post-surgery mice were administered ketoprofen 0.1 mg for pain control and were observed continuously for signs of pain or distress (hypoactivity, restlessness, vocalization, hiding, lack of grooming, abnormal posture, tremor, or respiratory distress) until they recovered from anesthesia, then monitored daily for 48 hr for signs of pain or distress. Humane endpoints were observed throughout experiments with mice being sacrificed when tumors reached a volume greater than 1500 mm^3^ by MRI assessment or when mice developed 15% weight loss. Mice were sacrificed via isofluorane anesthesia followed by cervical dislocation.

Human tumors were surgically implanted onto the pancreata of mice immediately following resection from either a patient (F_0_) or earlier generation xenograft (F_1_, F_2_, F_3_, etc). A 1.5-cm left flank incision was used to access the peritoneum of anesthetized mice, the pancreas was exteriorized using a sterile cotton swab and a small piece (∼25 mm^3^) of fresh patient tumor (F_0_ tumor) was sutured onto the pancreas using 5-0 Prolene (Ethicon, Cornelia, GA). The pancreas was repositioned and the wound closed using 4-0 Vicryl suture (Ethicon).

### Quantification of *in vivo* Tumor Growth and Metastasis

Tumor size was determined *in vivo* by volumetric magnetic resonance imaging (MRI). For MRI, mice were anesthetized and 0.5 mm axial imaging slices were generated, encompassing the entire tumor. Tumor area was measured for each individual image slice and tumor volume was calculated using the following: Volume_TUMOR_ = (Area_IMAGE1_+Area_IMAGE2_+Area_IMAGE3_…) as described previously [4]. Metastasis was assessed at necropsy by determining the percent of mice with grossly evident peritoneal and liver metastases.

### Obtaining Tumor Samples from Mouse Xenografts

Xenograft tumors were allowed to grow to a size of approximately 400–500 mm^3^ as measured by MRI at which point the mice underwent necropsy with complete tumor extirpation. Fresh tumor samples (approximately 50–60 mm^3^) were either: preserved in Allprotect (Qiagen, Valencia, CA) and stored at −80°C, snap-frozen in liquid nitrogen and stored at −80°C, cryopreserved in FBS with 10% DMSO; or fixed in formalin for subsequent analysis. Small pieces (∼25 mm^3^) were placed freshly in RPMI for reimplantation into the next generation of mice.

### Pathologic Assessment of Tumor Samples

Tumor pieces were placed in tumor blocks, fixed in zinc-buffered formalin for 24 hours, embedded in paraffin, and stored at −80 C. Hematoxylin/eosin staining was performed on human (F_0_) and mouse (F_1_, F_2_, etc.) tumors. A board-certified pathologist specializing in pancreatic and liver pathology (EBS) reviewed all slides, assessing tumor architecture and desmoplastic content and made qualitative comparisons between human and mouse tumors. Tumors were defined as having “abundant” desmoplasia when stroma comprised more than 50% of the cross-sectional tumor area.

### Genetic Mutation and Gene Expression Profiling

DNA was extracted from cell pellets using the PureLink™ Genomic DNA kit (Life Technologies, Grand Island, NY), and from frozen tissue samples preserved in Allprotect tissue reagent using the Allprep kit (Qiagen). DNA was quantified using a spectrophotometer and diluted to 50 ng/µl for PCR amplification, using Bio-X-Act™ short mix (Bioline, Taunton, MA) in a Techgene (Techne, Burlington, NJ) or Mastercycler gradient (Eppendorf, Hauppauge, NY) machine. The presence of mutation was assessed for the following genes: *KRAS*, *P53*, *SMAD4*, *BRAF*, and *BRCA2*. PCR primers and programs were based on published protocols and optimized for specific gene analysis [19], [20], [21], [22], [23], [24], [25]. PCR product quality was visualized by agarose gel analysis and purification was accomplished using the PureLink PCR purification kit (Invitrogen) or QIAquick Spin kit (Qiagen). Sequencing was performed by Genewiz Inc. (South Plainfield, NJ) or the University of Virginia Biomolecular Research Facility and analyzed using ApE plasmid editor v2.0.30 (M. Wayne Davis, University of Utah, Salt Lake City, Utah) or Vector NTI software (Life Technologies). DNA sequencing results were submitted to GenBank (Submission #1641200).

For gene expression profiling, tumor xenografts were analyzed and compared to original patient tumors (F_0_), normal human pancreas and four established and previously described [6] pancreatic cancer cell lines (Panc1, L3.6pl, BxPC-3 and MPanc96). The human pancreatic cancer cell line L3.6pl was kindly provided by I.J. Fidler (The University of Texas M.D. Anderson Cancer Center, Houston, TX; August 2005) [26]. MPanc-96, Panc-1, and BxPC-3 were obtained from the American Type Culture Collection in (Rockville, MD, August 2005) and maintained in DMEM (MPanc-96, Panc-1) or RPMI (BxPC-3) supplemented with 10% FBS and antibiotics. All cell lines were expanded, aliquoted and frozen upon initial receipt; cells were thawed, propagated and used for experiments every six months. MPanc-96, Panc-1 and BxPC-3 were authenticated prior to purchase by the ATCC with cytochrome c oxidase subunit 1 (COI) analysis, DNA profiling, cytogenetic analysis, flow cytometry, and immunocytochemistry. L3.6pl cells were authenticated in 2010 and 2011 by the UVABRF with DNA profiling, cytogenetic analysis, flow cytometry and immunocytochemistry. RNA was extracted from samples using the RNAeasy kit (Qiagen). Allprotect (Qiagen) was necessary for the preservation of RNA in all tissue samples. A TissueLyzer LT (Qiagen) was used to homogenize tissue and RNA was extracted using the Allprep kit (Qiagen). Processing of gene expression using the Affymetrix GeneChip system (Affymetrix, Santa Clara, CA) was provided by the UVABRF.

Gene expression was analyzed with unsupervised clustering methods. Using the “affy” package [27] and R/Bioconductor software tools [28], raw intensity values were normalized with robust multi-array averaging [29] and log-transformed to perform subsequent statistical analysis based on a Gaussian distributional assumption. A hierarchical clustering analysis with the Euclidian distance metric was used to generate agglomerative clusters with average linkage. Hierarchical clustering results were visualized as a dendrogram where closer branches represent samples with similar gene expression. In order to assess the consistency and preservation of molecular expression of our xenograft model, we performed genome-wide expression comparison based on a Pearson correlation analysis. The data presented in this publication have been deposited in NCBI's Gene Expression Omnibus and are accessible through GEO Series accession number GSE46385.

### Phospho-Receptor Tyrosine Kinase and Cytokine Arrays

Proteome Profiler™ Antibody Arrays (R&D Systems, Minneapolis, MN), including human phospho-receptor tyrosine kinase (RTK) and cytokine arrays, measured the activity of various signaling pathways. Lysates were prepared using NP-40 lysis buffer with leupeptin, aprotinin, sodium orthovanadate, and EDTA. Total protein concentration was determined using the Pierce BCA Protein Assay (Thermo Fisher Scientific, Inc., Waltham, MA), using 250 µg of lysate for each array. Arrays were developed using Pierce ECL Substrate (Thermo Fisher Scientific, Inc.) and analyzed using a Bio-Rad GS-800 densitometer (Bio-Rad, Hercules, CA) and ImageQuant TL 2005 (GE Healthcare, Piscataway, NJ) software.

Phospho-RTK and cytokine expression were normalized to the maximum value of the negative control for each array as follows. The final value was calculated by determining the average density of each sample and subtracting the standard error of the mean and the maximum value of the negative controls (i.e. background). Data are reported as multiples of background signal for each receptor or cytokine.

### Data Analysis and Statistical Methods

For statistical analyses, group comparisons were unpaired. Categorical variables were compared using Fischer's exact, McNemar's, or Pearson's Chi square tests, as appropriate. Analysis of variance compared continuous variables. Categorical variables are expressed as percentages of the group of origin, and continuous variables as means ± standard deviation. Survival curves were generated using the Kaplan-Meier method and analyzed using the log-rank test. All *p*-values are two-tailed, and significance indicated by *p*-values<0.05. Association between time to establishment of F_1_ tumors in mice and patient survival time was analyzed with a non-parametric Spearman rank-order correlation analysis. GraphPad Prism software (La Jolla, CA) and R statistical software (R Foundation for Statistical Computing, Vienna, Austria) were used for all statistical analyses.

## Results

### Model Overview

To date, 47% (21/45) of initially implanted tumors (F_1_) have successfully grown in mice; of which greater than 95%, were also successfully propagated in subsequent generations (F_2_, F_3_, …) of mice (data not shown). Patient tumors (F_0_) derived from metastatic patient lesions were more likely to grow (7/8, 88%) compared to patient-derived primary pancreatic tumors (14/37, 38%; *p* = 0.01); the former were established more quickly (3.4±0.3 months vs. 5.1±0.5 months, *p* = 0.02), and were associated with increased incidence of liver and peritoneal metastasis in F1 mice (67% vs. 31%, *p* = 0.002).

**Figure 2:**
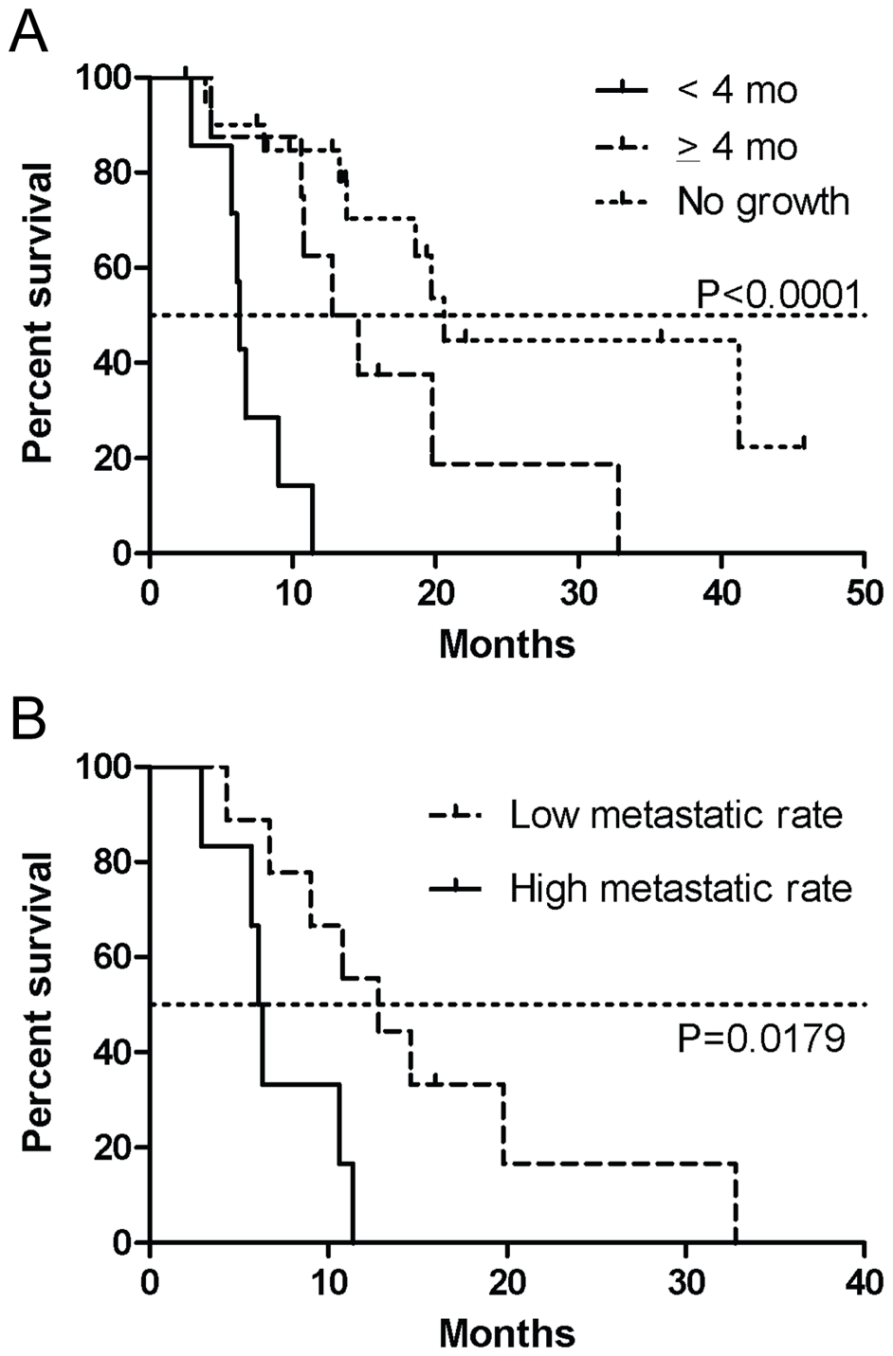
Growth rate and metastatic rate of xenografts predicts patient survival. Kaplan-Meyer curves of patient survival based on rate of tumor growth (A), and metastasis (B) in F_1_ mice.

Time to establishment of F_1_ tumors in mice significantly correlated with patient survival for all patient tumors engrafted (Spearman rank-order correlation coefficient = 0.36, *p* = 0.034) and, strikingly, more highly correlated for successfully propagated tumors (Spearman rank-order correlation coefficient = 0.56, *p* = 0.031). Patients whose F_1_ tumors grew to 400–500 mm^3^ in less than four months after implantation had a median survival of 6.3 months, compared to 13.7 months when F_1_ tumors took 4 months or longer to grow, and 20.6 months when tumors failed to grow (*p*<0.0001, Figure 2A). Additionally, the rate of peritoneal and liver metastasis in F_1_ mice correlated with patient survival (Figure 2B). Patients whose F_1_ tumors developed peritoneal and liver metastasis rates of >50% in mice had a median survival of 6.5 months whereas patients whose tumors had a metastasis rate of ≤50% in mice had a 13 month median survival. (*p* = 0.0179).

### Pathologic Comparison

**Figure 3:**
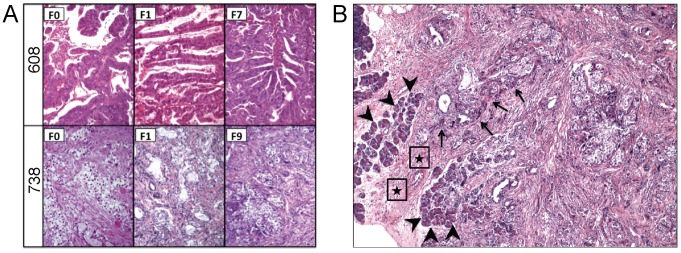
Pathological comparisons of human and mouse tumors. Hematoxylin and eosin (H&E) staining (100×) of human (F_0_), early (F_1_), and late (F_7_, F_9_) passage mouse tumors are shown (A), demonstrating preserved histologic architecture and stroma. H&E staining (200×) of a surgically implanted human tumor in the mouse pancreas (B); large arrowheads demonstrate normal pancreatic lobules, stars show interlobular fibrosis/chronic pancreatitis near the invasive tumor front, and smaller arrows demonstrate invading cancer cells.

Neither histological differentiation (McNemar's *p* = 0.48) nor stromal content (McNemar's *p* = 0.25) differed significantly between mouse xenograft tumors and their respective F_0_ patient tumors (Table 1). Seven tumors were graded as “poorly differentiated” for both F_0_ and F_1_ tumors; 3 were graded as well to moderately differentiated for both F_0_ and F_1_ tumors; and 5 tumors were graded discordantly between F_0_ and F_1_ tumors. Stromal content was consistent in both F_0_ and F_1_ specimens for 13 of 15 tumors (9 exhibited a high stromal content in both; 3 exhibited a low stromal content in both), while two tumors had low stromal content in F_0_ generation, but exhibited a higher level of stroma in F_1_. Both differentiation state and stromal content was preserved among early as well as late passage mouse tumors (Figure 3A). Xenografted tumors demonstrated infiltration of cancer cells into the normal mouse pancreata, obliteration of normal pancreatic lobules by cancer cells, and desmoplasia (Figure 3B). Metastatic sites in mice bearing pancreatic xenografts were similar to that seen in humans (liver, diaphragm, and peritoneum). Retroperitoneal invasion commonly occurred in mice, similar to invasive patterns in humans (data not shown).

**Table 1:** Clinical and pathological comparisons of human and mouse tumors.

Tumor	Overall Stage	T Stage	F_1_ Time to Growth (mo)	F_0_: Primary or Met; F_1_ Met Rate		Differentiation^1^		Tumor Stromal Content	
	F_0_	F_0_	F_1_	F_0_	F_1_	F_0_	F_1_	F_0_	F_1_
608	IV	2	2.0	Met	100%	Well-Mod	Well-Mod	Low	Low
738	Ib	3	7.2	Primary	25%	Poor	Poor	High	High
232	IIb	2	3.9	Primary	25%	Poor	Poor	High	High
366	IV	2	2.8	Met	75%	Poor	Poor	Low	Low
431	IIb	1	6.2	Primary	25%	Poor	Poor	High	High
432	IV	3	3.3	Met	29%	Poor	Poor	High	High
530	IIb	2	4.3	Primary	0%	Well-Mod	Poor	High	High
1049	IIb	3	7.3	Primary	0%	Well-Mod	Well-Mod	High	High
215	IV	3	3.8	Met	75%	Mod-Poor	Poor	Low	High
450	IIb	3	5.5	Primary	100%	Well-Mod	Poor	Low	Low
602	IV	3	3.9	Met	0%	Mod-Poor	Well-Mod	High	High
624	IIb	3	4.8	Primary	25%	Poor	Poor	High	High
653	IIb	3	4.5	Primary	50%	Well-Mod	Well-Mod	Low	Low
654	IV	2	3.3	Met	83%	Well-Mod	Poor	Low	High
912	IIb	3	1.7	Primary	55%	Poor	Poor	High	High

1 Well-Mod: mild to moderate cytologic atypia, gland forming growth; Poor: marked cellular atypia, solid and single cell growth.

### Genetic Mutations

**Figure 4:**
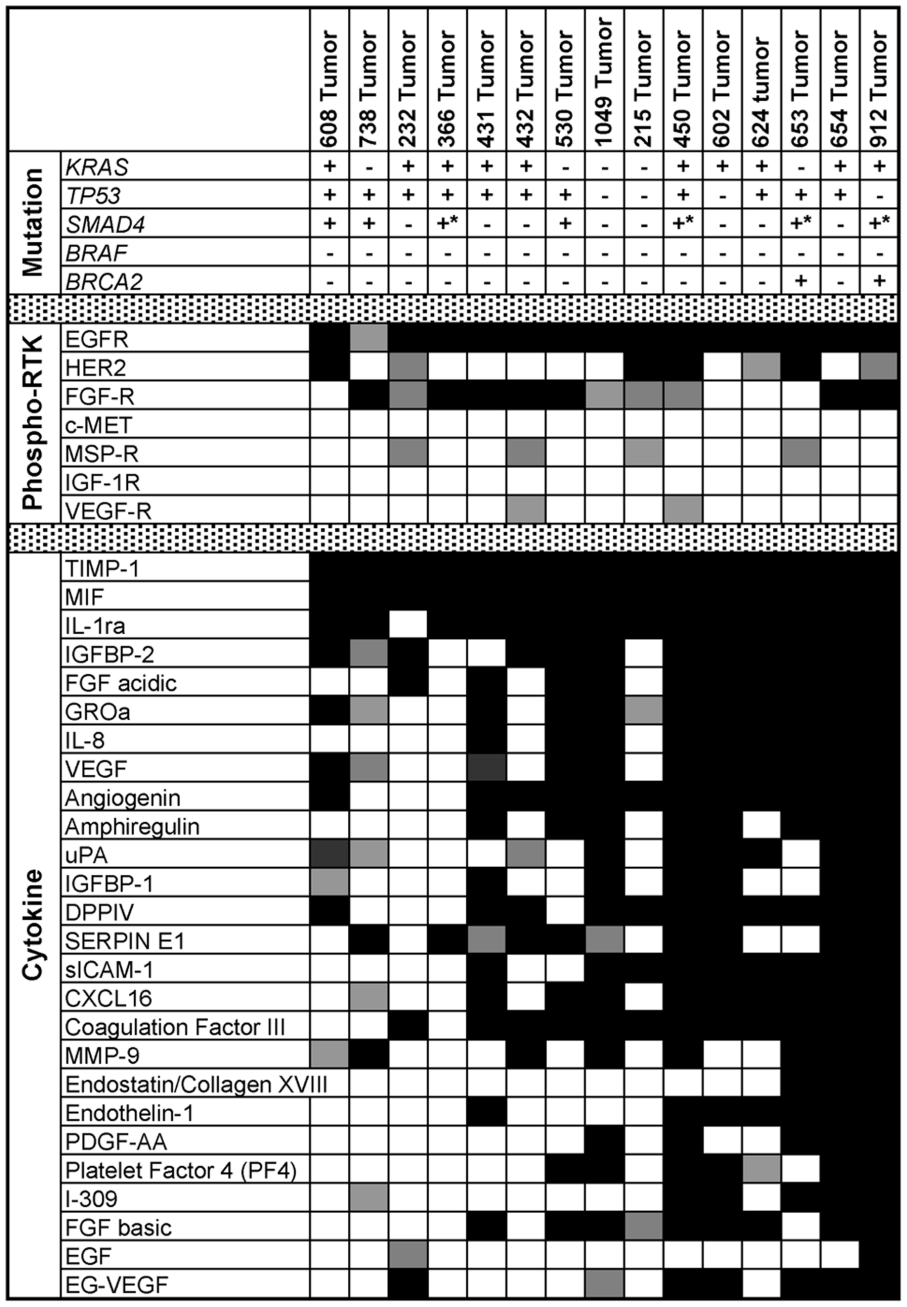
Genetic and molecular features of the human tumors propagated in mice. (A) Genetic mutational status of the PDAC tumors; (+) indicates a mutation while (−) indicates wild-type status. For the SMAD4 analysis (*) denotes tumors with rearranged or deleted sequences within the analyzed exons (e.g., 9, 10 and 11) indicating mutations within the coding regions of the gene. Ranked threshold heat maps of RTKs that are activated (phosphorylated) (B) and cytokines that are upregulated (C) in xenografted PDACs. Squares represent activation of RTKs over threshold (Black: >3 times threshold, Dark gray: 2–3 times threshold, Light gray: 1–2 times threshold, White: no increase above threshold).

PCR amplification and DNA sequencing of DNA from 15 individual F_1_ tumors was carried out to assess common sequence alterations present in PDACs (Figure 4, top panel). *KRAS* mutations were observed in 10/15 tumors (67%) with all mutations occurring in codon 12 and none in codons 13 or 61. *p53* mutations were present in 11/15 tumors (73%), involving various codons within exons 5, 6, and 8 [22]. *SMAD4* mutations were present in 7/15 tumors (47%), involving sequence alterations in exons 8, 9, 10, or 11. No *BRAF* mutations were observed. *BRCA2* missense mutations were observed in 2 of 15 tumors (13%).

### Phospho-RTK Activation and Cytokine Expression

Whole tumor lysates from F_1_ tumors were assessed for activation (phosphorylation) of 47 RTKs (Figure 4, middle panel). All tumors demonstrated activation of epidermal growth factor receptor (EGFR). Human epidermal growth factor receptor 2 (HER2/ErbB2) was activated in 7/15 tumors (47%), and fibroblast growth factor receptors 1 and 3 (FGFR 1/3) were activated in 10/15 tumors (67%). Other RTKs, such as macrophage stimulating protein receptor (MSPR/RON) and vascular endothelial growth factor receptor (VEGF-R) were activated in four (27%) and two (13%) tumors, respectively. No tumor exhibited activation of hepatocyte growth factor receptor (c-MET) or insulin-like growth factor 1 receptor (IGF-1R). Tumors displayed significant variability in human cytokine production (Figure 4, bottom panel). Notably, EGF family ligands (EGF and amphiregulin), FGF (acidic and basic) and VEGF were present, consistent with the observed autocrine activation of these receptors in some tumors. Furthermore, well to moderately differentiated tumors expressed a greater number of cytokines (19.9 vs. 12.6, *p* = 0.0423) and higher concentrations of cytokines (those with >3-fold threshold: 19.1 vs. 11.0, *p* = 0.0382) compared to poorly differentiated tumors, again emphasizing the importance of studying genetically diverse human tumors and pointing to the influence of differential gene expression on the tumor cell-microenvironment interactions.

### Gene Expression

**Figure 5:**
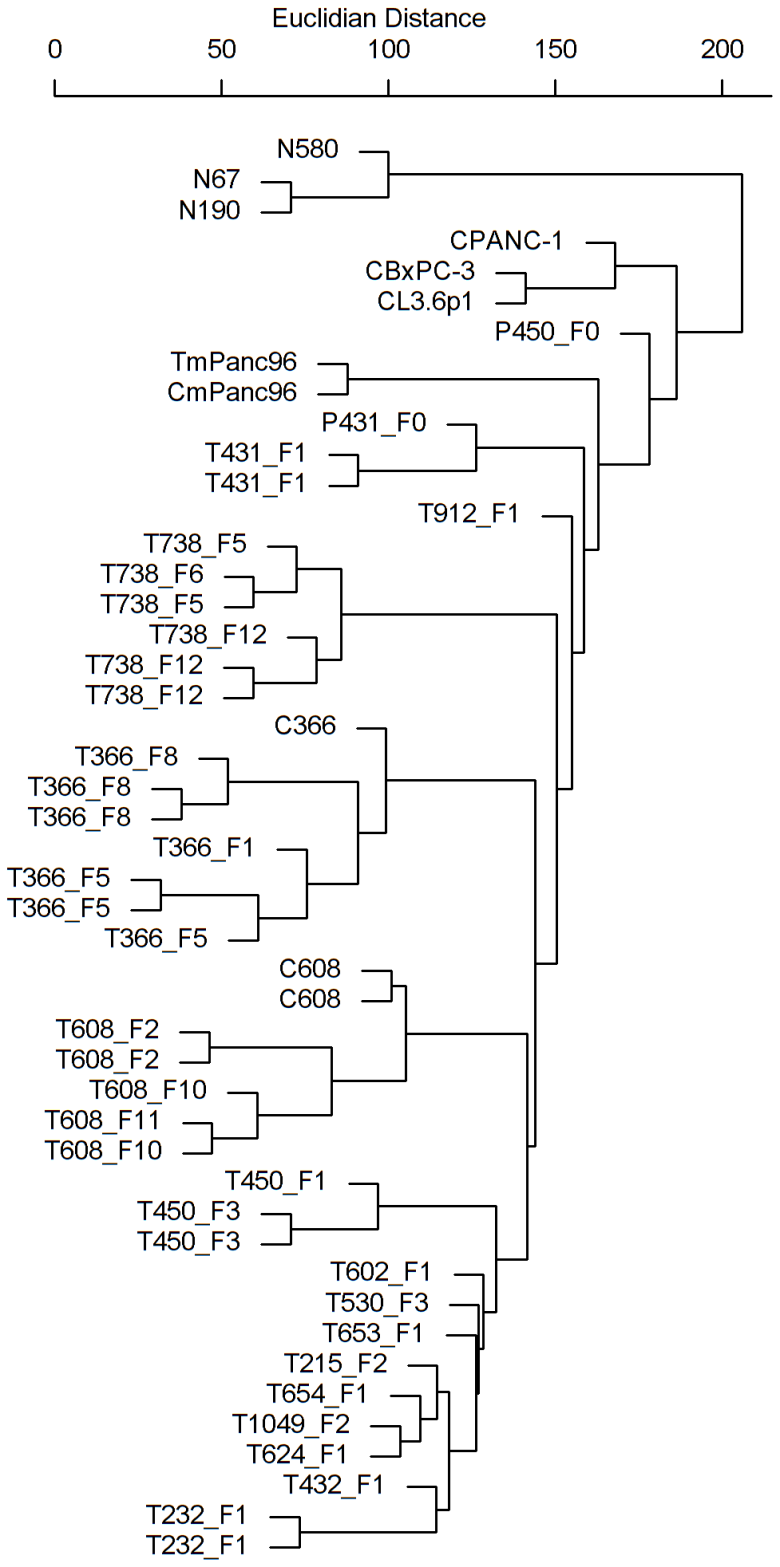
Comparison of gene expression profiles for human tumors and mouse xenografts. Unsupervised clustering of gene expression profiles for three normal pancreatic specimens (N580, N190, N67), four pancreatic cancer cell lines (BxPC-3, L3.6pl, PANC-1, MPanc96) and each orthotopically xenografted human PDAC. A hierarchical clustering analysis with the Euclidian distance metric was used to generate agglomerative clusters with average linkage. Hierarchical clustering results are visualized as a dendrogram where closer branches represent samples with similar gene expression. Cell lines are denoted with “C” prefix and tumor lysates denoted with “T” prefix.

Gene expression profiling was performed for xenografted tumors, established cell lines, and normal human pancreatic specimens. Unsupervised clustering is shown as a dendrogram in Figure 5. Normal pancreatic specimens demonstrate similar gene expression patterns and cluster distinctly from both cancer cell lines and xenografts. The majority of the previously established, high passage pancreatic cancer cell lines (L3.6pl, BxPC-3, and PANC-1) clustered together, distinct from patient xenografts, the exception being the MPanc96 tumor. This suggests that established, commercially available, high-passage PDAC cell lines differ significantly in gene expression from fresh human PDAC specimens and emphasizes the need to use fresh human PDAC specimens in animal models. However, a caveat of our data analysis is that cell lines only (and not xenografted tumors) were analyzed for L3.6pl, BxPC-3 and PANC-1. Figure 5 also demonstrates that early and later passages of the same human tumor clustered together as do low passage cell lines derived from the xenografts. Also, noted in the dendrogram are clusters of tumors which demonstrate greater similarity in gene expression as compared to others. For example, of the 7 tumors which cluster to the far right in Figure 5, four of 7 demonstrate MSP-R (RON) activation (Figure 4, middle panel) compared to 0 of 8 tumors in the left portion of the dendrogram (*p* = 0.0256).

**Figure 6:**
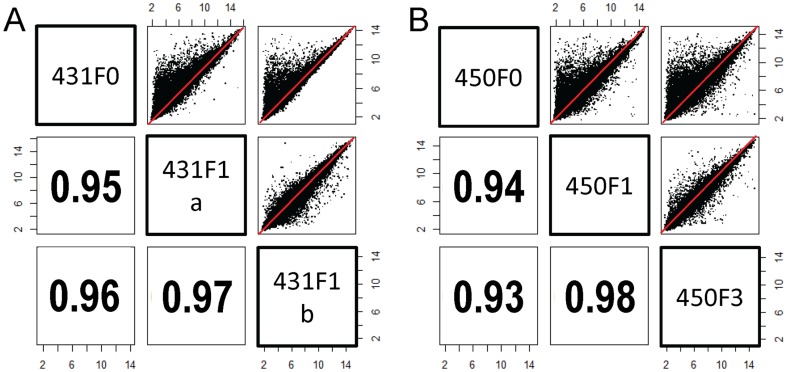
Genome-wide expression comparison analysis. Gene expression patterns on >40,000 probe sets were plotted among xenograft models both from the same and independent patient tumors. Performing genome-wide expression comparison analysis based on Pearson correlations, we found the xenograft models from the same patient tumors showed significantly more consistent expression patterns than those from different patient tumors. Data are shown for tumor 431 (A) and 450 (B). Numbers represent Pearson correlation coefficient between tumor sample above and to right of number.

Comparison of genome-wide gene expression in the human tumors (F_0_) with xenografted tumors (F_1_ to F_7_) demonstrated significantly higher correlations in gene expression across ∼>40,000 probe sets with the average (Pearson) correlation coefficient 96% (93∼98%) than comparison of two independent xenograft models with the average correlation 93% (89∼95%) (Student t-test *p*-value<0.01; Figure 6).

## Discussion

The current literature contains numerous preclinical studies demonstrating therapeutic efficacy in PDAC, but these have failed to translate into successful clinical trials [30], [31]. To improve outcomes for PDAC, novel therapeutic strategies are needed, along with improved understanding of how tumors adapt to and become resistant to therapeutic treatments. Thus, preclinical models that closely recapitulate human PDAC are necessary. Unfortunately, no perfect model exists, and all models have inherent limitations. An ideal PDAC model would: (1) be efficiently established and easily propagated, (2) accurately reflect human tumor features and heterogeneity, (3) mimic human metastatic patterns, (4) possess a relevant tumor microenvironment, and (5) have limited “drift” through subsequent passages. Guided by these principles, we have described and validated a murine, orthotopic xenograft model of human PDAC using fifteen fresh human derived tumors.

Efficient establishment and propagation of tumors is essential in any xenograft model. In the model described here, nearly half of original F_0_ tumors grow in F_1_ mice (generally within two to six months) and greater than 95% grow in subsequent generations. Interestingly, the time to initial tumor engraftment to a size of 400–500 mm^3^ in the mouse pancreas correlated with patient survival. In addition, patient-derived metastatic tumors were more likely to grow in F_1_ mice. This suggests that more aggressive patient tumors also grew more aggressively as mouse xenografts in this model. This parallels studies which demonstrated that patients whose non-small cell lung cancers successfully engrafted into mice had significantly shorter disease free survival, compared to patients whose tumors did not establish [32].

To be adequate representations of human cancers, xenograft models must accurately reflect the histopathologic and molecular features as well as the diversity of human tumors. In the model described above, orthotopically propagated mouse tumors mimic the architecture and stromal content of their respective human tumors, maintaining tumor grade through multiple passages, similar to previous observations in lung and breast cancer models [33], [34]. Perhaps, the most important feature of preclinical tumor models is the faithful maintenance of gene expression signatures between the original patient tumor and subsequent mouse xenografts. Affymetrix based gene expression profiling revealed a high degree of conservation of gene expression with correlation coefficients of 93% to 99% when individual patient tumors (F_0_) were compared to F1 tumors and subsequent tumors passaged through multiple generations. However, one limitation of our approach is the lack of comparison of the tumors to a larger dataset containing unmatched patient tumors and mouse xenografts. Despite this limitation, to our knowledge, this is the first preclinical pancreatic cancer model to display such highly conserved gene expression. The diversity of oncogenic drivers of tumor progression is one of the greatest strengths of the xenograft model and one of the key limitations of engineered cancer models. Patient derived xenografts exhibit multiple, well studied genetic mutations common to human PDACs — *KRAS*, *P53*, and *SMAD4* [35], [36], [37]. The mouse xenografts demonstrated activation of multiple RTKs, notably EGFR and Her2, which are relevant targets in human PDAC [38], [39], [40], [41]. The conserved patterns of RTK activation for individual tumors provide an opportunity to assess different therapeutic strategies, choosing relevant targets and customizing therapy based on the unique features of a particular tumor and identifying biomarkers of response to therapy [6].

Because metastatic lesions provide the greatest therapeutic challenge and impact on survival, an ideal tumor model should recapitulate human metastatic patterns. While subcutaneous injection and spontaneous tumor models are limited or variable in this regard [7], we observed that mice bearing pancreatic xenografts frequently develop liver, diaphragmatic, and peritoneal metastases, with local retroperitoneal invasion. This xenograft model allows the comprehensive investigation of genetic and molecular pathways that drive metastatic disease as well as directly test new therapeutic strategies targeting metastasis.

The impact of the tumor microenvironment cannot be overemphasized, as it influences drug delivery, is an important source of tumor growth factors and contributes to tumor survival in the face of therapeutic treatments. The cytokine array data (Figure 4, bottom panel) demonstrating production of numerous stromal-interacting cytokines (e.g. TIMP-1, MIF, uPA, Serpin E1/PAI-1, MMP-9) suggests that the xenografts are interacting with and being altered by components of the tumor microenvironment. Investigating tumors within a relevant microenvironment provides study of cancer cell-stromal interactions, and uncovers potential therapeutic targets within the microenvironment. For example, Olive et al. demonstrated that targeting the PDAC microenvironment improves drug delivery and survival [42]. We recently reported that inhibition of RAS pathway signaling with trametinib, an inhibitor of MEK1/2, blocked pancreatic cancer cell proliferation in a variety of cell lines tested [6]. We also noted that the combined inhibition of EGFR/HER2 with the EGFR/HER2 inhibitor lapatinib failed to substantially inhibit cell proliferation in cultured cell lines whereas when assessed in the orthotopic xenograft model, treatment with lapatinib and trametinib resulted in significantly enhanced inhibition of tumor growth relative to trametinib treatment alone. Molecular analysis of drug treated orthotopic tumors provided evidence that inhibition of MEK1/2 signaling leads to the feedback upregulation of RTK signaling and activation of cell survival pathways in tumors growing orthotopically in the pancreas. Such compensatory signaling was not observed in cell culture, underscoring the importance of assessing drug responses in the context of an appropriate microenvironment. In a related study we investigated the effects of the focal adhesion kinase (FAK) inhibitor (PF-562,271) on pancreatic cancer and stromal cell migration *in vitro* and assessed its effects on tumor growth and metastasis in an orthotopic murine model [4]. We reported that PF-562,271 effectively inhibits both pancreatic cancer cell and stromal cell migration and invasion in *in vitro* cell culture assays. Treatment of orthotopic tumor bearing mice with PF-562,271 leads to a significant inhibition of tumor growth and reduces the number of tumor associated stromal cells, indicating that inhibition of adhesion signaling reduces pancreatic cancer cell growth either directly by contributing to inhibition of cell proliferation and/or by altering the cellular composition of the tumor microenvironment. Thus, the use of preclinical tumor models with a relevant tumor microenvironment will be essential in making progress in cancer therapeutics.

While no perfect model exists for PDAC and important data may be garnered from each model, the orthotopic xenograft model using a diverse array of freshly implanted human tumors provides a unique platform for assessing tumor responses to therapeutic strategies. The orthotopic xenograft model using fresh human specimens most closely recapitulates the molecular and genetic heterogeneity of PDAC, human metastatic patterns, the tumor microenvironment, and drug delivery. This model holds great promise for identification and testing of additional rational therapeutic approaches for pancreatic cancer and the selection of personalized cancer therapies, and provides the opportunity for the identification of genetic and molecular biomarkers of response to therapy.
